# Gold Nanoparticles Synthesized Using Extracts of *Cyclopia intermedia*, Commonly Known as Honeybush, Amplify the Cytotoxic Effects of Doxorubicin

**DOI:** 10.3390/nano11010132

**Published:** 2021-01-08

**Authors:** Jumoke A. Aboyewa, Nicole R. S. Sibuyi, Mervin Meyer, Oluwafemi O. Oguntibeju

**Affiliations:** 1 Phytomedicine and Phytochemistry Group, Oxidative Stress Research Centre, Department of Biomedical Sciences, Cape Peninsula University of Technology, Bellville 7535, South Africa; Jumokeaboxe@gmail.com; 2 DSI/Mintek Nanotechnology Innovation Centre, Biolabels Node, Department of Biotechnology, University of the Western Cape, Bellville 7530, South Africa; nsibuyi@uwc.ac.za

**Keywords:** *Cyclopia intermedia*, honeybush, green synthesis, gold nanoparticles, mangiferin, nanotechnology

## Abstract

*Cyclopia intermedia* (*C. intermedia*) is an indigenous South African shrub used to prepare the popular medicinal honeybush (HB) tea. This plant contains high levels of mangiferin (MGF), a xanthonoid that was reported to have numerous biological activities, including anti-tumor activity. MGF and extracts that contain high concentrations of MGF, such as extracts from *Mangifera indica* L. or mango have been used to synthesize gold nanoparticles (AuNPs) using green nanotechnology. It has previously been shown that when AuNPs synthesized from *M. indica* L. extracts are used in combination with doxorubicin (DOX) and Ayurvedic medicine, the anti-tumor effects appear to be augmented. It has also been demonstrated that MGF used in combination with DOX resulted in enhanced anti-tumor effects. In this study, *C. intermedia* (HB) and MGF were used to synthesize HB-AuNPs and MGF-AuNPs, respectively. The physicochemical properties of the AuNPs were characterized by the UV-Visible Spectroscopy (UV-Vis), dynamic light scattering (DLS), Fourier transform infra-red spectroscopy (FTIR), X-ray diffraction spectroscopy (XRD) and high-resolution transmission electron microscopy (HR-TEM). The cytotoxicity of HB-AuNPs and MGF-AuNPs were assessed on human colon (Caco-2), prostate (PC-3) and glioblastoma (U87) cancer cells; as well as normal breast epithelial (MCF-12A) cells using the MTT assay. Both HB-AuNPs and MGF-AuNPs demonstrated relatively low cytotoxicity in these cells. However, when these nanoparticles were used in combination with DOX, the cytotoxicity of DOX was significantly augmented.

## 1. Introduction

Several studies have reflected on the bio-activity of mangiferin (MGF), the xanthonoid known for the treatment of several health problems not limited to diabetes [1], inflammation, obesity, cancer [1,2] and osteoarthritis [2,3]. The health benefits of MGF have prompted a rigorous research to find other sources of this valuable compound [4,5]. While the extracts from mango (*Mangifera indica* L.) fruit peel [6], seeds, leaves [2,7,8], bark and roots [2,7] have been shown to contain this compound, MGF is also found abundantly in other plant species such as *Salacia chinensis*, *Swertia chirata*, *Hypericum aucheri* and *Cyclopia intermedia* [6,9,10]. The xanthones (MGF and isomangiferin) and flavanones (hesperitin and hesperidin) are the major components of *C. intermedia* extracts, and are thought to be responsible for most of its pharmacological effects, including anti-diabetic, anti-cancer, anti-obesity, antioxidant and antimicrobial activities [11,12].

Independent studies have reported that MGF has excellent anti-cancer effects, however its exact molecular mechanism has not been fully elucidated. Previous evidence clearly indicates that MGF can inhibit the proliferation of cancer cells via the induction of apoptosis, and its ability to regulate apoptotic pathways via multiple targets has been documented [13]. A study by Zou et al. showed that MGF induced apoptosis of A459 human lung cancer cells by activating NF-κB and caspase-dependent pathways [14]. Pan et al. reported the suppression of Bcl-xL and XIAP expression and the blocking of nuclear entry of NF-κB in HL-60 human acute myeloid leukemia cells treated with MGF [15].

It has also been reported that the co-administration of MGF and the chemotherapeutic drug doxorubicin (DOX) can promote the anti-cancer effects of DOX in U-937 human myeloid leukemia cells [16]. Another study showed that MGF sensitized the response of MCF-7 breast cancer cells to DOX by suppressing the expression of p-glycoprotein [17]. It is also believed that MGF might be a promising agent to reverse DOX resistance in cancer cells and is thus considered as a potential chemosensitizer for DOX therapy [17].

Numerous studies reported on the use of green nanotechnology to synthesize AuNPs from *M. indica* L. [18,19,20] and MGF [6,9]. While MGF is a major phytochemical constituent of the peel of *M. indica* L., it is not known if MGF is involved in the synthesis of the AuNPs when synthesis is done using a plant extract. Al-Yasiri et al. reported the synthesis of radiolabelled gold nanoparticles (AuNPs) (MGF-^198^AuNPs) using MGF. The study also showed that MGF-^198^AuNPs were retained in prostate tumors, resulting in a significant reduction of tumor size [6]. It was also recently demonstrated that Nano Swarna Bhasma, AuNPs synthesized using a mixture of plant phytochemicals isolated from *M. indica* L. peel and proprietary combinations of *Emblica officinalis*, *M. indica* L., *Curcumin longa*, *Acacia nilotica* and *Glycyrrhiza glabra*, was highly toxic to the MDA-MB-231 breast cancer cells [9]. Experiments using Severe Combined ImmunoDeficient (SCID) mice showed that this treatment (Nano Swarna Bhasma) reduced breast tumor sizes in these animals. Importantly, the study also showed in a pilot human clinical investigation that breast cancer patients who received the Nano Swarna Bhasma treatment in addition to conventional anti-tumor drugs (DOX and cyclophosphamide) experienced significant therapeutic benefits [9].

In the present study, we investigated if AuNPs can be synthesized using water extracts produced from the *C. intermedia*, which also has a high MGF content. *C. intermedia*, commonly known as honeybush (HB), is indigenous to the southwestern and southeastern parts of South Africa [21]. The leaves of HB are commonly used to make herbal tea, which has many health benefits. HB is rich in antioxidants and the health benefits include the treatment of infections, coughs, sore throat, colds, osteoporosis, prevention of cancer and asthma [11]. The genus *Cyclopia* consists of some 20 species of flowering plants in the legume family, Fabaceae [4]. Several species, which include *C. longifolia*, *C. subternata*, *C. sessiliflora*, *C. maculata*, *C. genistoides* and *C. intermedia,* grow naturally in the wild in various regions of South Africa. To our knowledge, this plant has not previously been used for the synthesis of AuNPs. The study demonstrated the synthesis of AuNPs using leaf extract of *C. intermedia*. We showed that these nanoparticles had low cytotoxicity towards the selected cells but appeared to significantly augment the cytotoxic effects of DOX in Caco-2 cells when used in combination with DOX.

## 2. Results

### 2.1. Biosynthesis of HB and MGF-AuNPs

HB is rich in polyphenols, of which MGF (62.721 mg/g) and hesperidin (40.742 mg/g) are the most abundant [22]. Although the synthesis of AuNPs has been reported for both MGF and hesperidin [6,10,23], MGF with its four hydroxyl groups and a glucose unit exhibits stronger bio-reduction of metal precursors to form nanoparticles. It is therefore likely that MGF found abundantly in *M. indica* L., and HB extracts (HBE) can act as both reducing and stabilizing agent during the synthesis of AuNPs. However, due to the complex and diverse nature of the phytochemicals present in plant extracts it is almost impossible to pinpoint a particular phytochemical responsible for the bio-reduction and stabilization of the nanoparticles. Of interest, phenolic compounds (caffeic acid, gallic acid) and flavonoids (anthocyanidins, isoflavones, flavones and flavols) were reported to play a major role in the synthesis of metallic nanoparticles [24,25]. This is due to the high nucleophilic character of hydroxyl and carbonyl groups as well as their excellent binding affinity to metal ions [26,27].

Polyphenolic compounds such as flavonoids and phenols are the major constituents of antioxidants in most plant species and their antioxidant activity is mainly credited to their redox properties. As a result, they can act as reducing agents by aiding in the reduction of metallic ions into nanoparticles [28]. It is known that plants with a higher total phenolic content (TPC) show high antioxidant activity and consequently possess excellent reductive capacity necessary for the synthesis of nanoparticles [29]. The phytochemical constituents, TPC and antioxidant capacity of HBE and MGF are compared and displayed in Table 1. MGF was commercially obtained; it was isolated from *M. indica* L. bark and had the purity of ≥98% (TLC). The comparative phytochemical analysis of HBE and MGF revealed that 10 mg/mL HBE contained high amounts of flavanols (8.38 mg/g), and they were not detected in the MGF. Flavonols were present in HBE (0.3 mg/g), but at a much lower concentration than in the MGF (8.7 mg/g). The TPC was also significantly higher in MGF (1.30 mgGAE/g) than in HBE (0.18 mgGAE/g). A previous study has shown that plants with high TPC show higher reducing capacities for nanoparticle synthesis. Goodarzi et al. reported the successful synthesis of nanoparticles from various plant extracts and reported that *Zataria multiflora* has the highest TPC and is thus more potent in nanoparticle synthesis [30]. The antioxidant activity of HBE as measured by 2,2-diphenyl-1-picrylhydrazyl (DPPH) radical scavenging, oxygen radical absorbance capacity (ORAC) and ferric reducing antioxidant power (FRAP) activity was also lower for HBE compared to MGF. Several plant extracts with high reductive capacity have been reported to actively reduce metallic ions, including silver and gold ions, to their corresponding metallic nanoparticles [31,32,33]. This reductive capacity can be easily traced to the secondary metabolite content of the plants, which includes polyphenols, flavonoids and steroids. Numerous studies have also demonstrated that these metabolites in the aqueous medium act as both reducing and stabilizing agents for the synthesis of metallic nanoparticles [34,35]. This study revealed that HBE exhibited high reductive capacity and, based on this, was likely to reduce gold ions to form AuNPs.

**Scheme 1:**
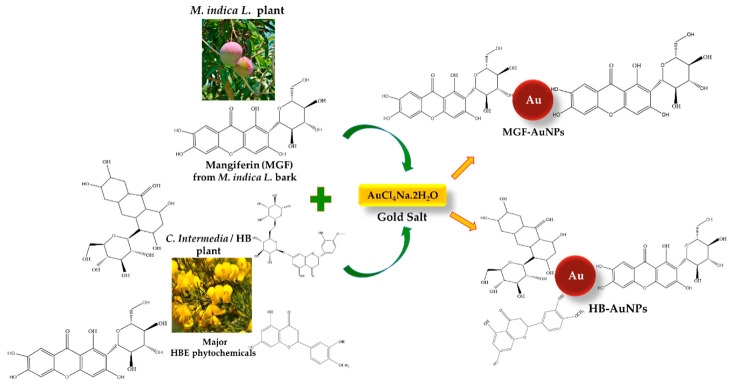
Possible mechanism for green synthesis of biogenic MGF-gold nanoparticles (AuNPs) and HB-AuNPs.

AuNPs were successfully synthesized using both HBE and MGF and the possible mechanism for AuNP synthesis is depicted in Scheme 1. During synthesis, a distinctive ruby red color change was observed within 10 min after sodium tetrachloroaurate (III) dehydrate and HBE were combined at 70 °C to produce HB-AuNPs. In comparison, the reaction with MGF was almost immediate (<2 min). For the synthesis of MGF-AuNPs, gum arabic (GA) was added to stabilize the nanoparticles. GA is a non-toxic glycoprotein polymer commonly used as a stabilizer in the food and pharmaceutical industries. It has been reportedly used to functionalize and stabilize AuNPs owing to the excellent binding affinity of its abundant carboxyl groups to other biomolecules. The introduction of GA resulted in a color change from brown (for MGF without GA) to a ruby red color that appeared immediately, indicating the formation of stable MGF-AuNPs.

#### Characterization of MGF-AuNPs and HB-AuNPs

**Figure 1:**
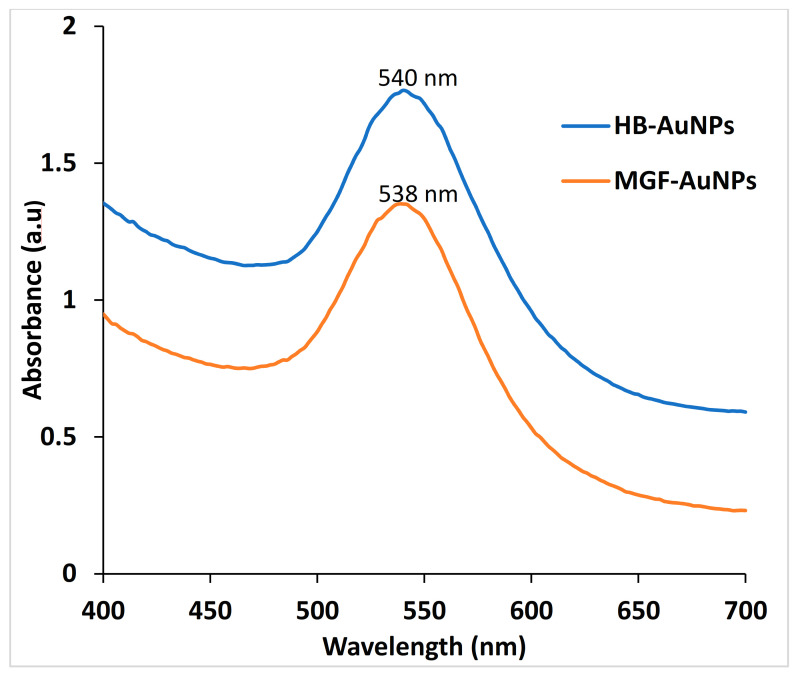
UV-Visible absorption of HB-AuNPs and MGF-AuNPs.

HB-AuNPs and MGF-AuNPs were characterized by UV-Visible spectrophotometry, dynamic light scattering (DLS) and high-resolution transmission electron microscopy (HR-TEM) analysis. HB-AuNPs and MGF-AuNPs produced maximum UV-Vis absorption peaks at 540 and 538 nm, respectively (Figure 1), which corresponds to the surface plasmon resonance (SPR) for the AuNPs.

The hydrodynamic diameter, polydispersity index (PDI) and zeta potential of the biogenic AuNPs were measured by DLS. As shown in Table 2, the z-average sizes of HB-AuNPs and MGF-AuNPs were similar. The diameters of HB-AuNPs and MGF-AuNPs were 66.74 ± 9.7 nm and 65.50 ± 15.15 nm, respectively. The zeta potential and PDI of HB-AuNPs were −23.45 ± 1.4 mV and 0.57 ± 0.01, respectively. MGF-AuNPs had a zeta potential of −27.87 ± 2.54 mV and a PDI of 0.43 ± 0.07. The characteristics of HB-AuNPs and MGF-AuNPs as measured by DLS were very similar. Nanoparticles with a zeta potential between −30 and +30 mV are stable and will not aggregate in solution [36]. This suggests that HB-AuNPs and MGF-AuNPs are stable in solution.

**Figure 2:**
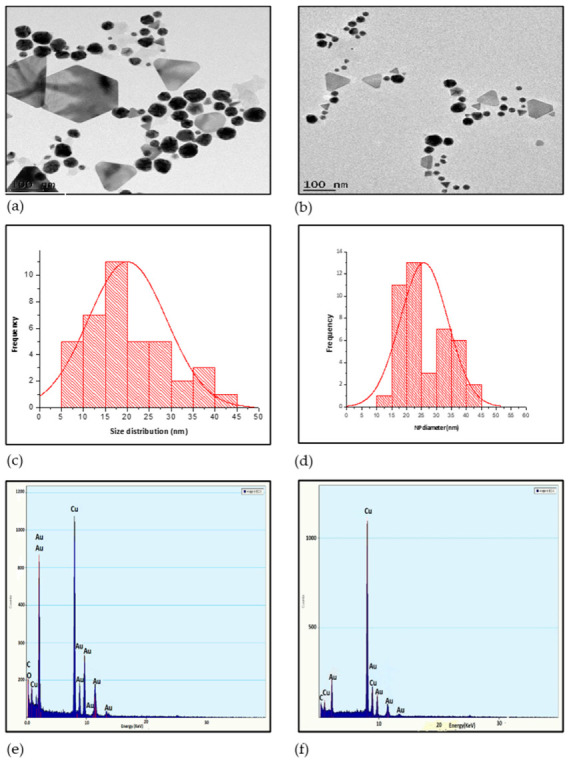
Morphology and energy dispersive X-ray spectroscopy (EDX) profiling of the biogenic AuNPs. High-resolution transmission electron microscopy (HR-TEM) micrographs of HB-AuNPs (**a**) and MGF-AuNPs (**b**); particle size distribution of HB-AuNPs (**c**) and MGF-AuNPs (**d**); EDX profiles of HB-AuNPs (**e**) and MGF-AuNPs (**f**) for their chemical composition.

HR-TEM image analysis showed similar characteristics between HB-AuNPs and MGF-AuNPs. Both HB-AuNPs and MGF-AuNPs are polydisperse with most nanoparticles having predominantly spherical shapes and some triangular shapes. The core diameter of the nanoparticles varied from 5 to 45 nm (Figure 2a,b). The average core size for HB-AuNPs was 20 nm (Figure 2c) and 26 nm for MGF-AuNPs (Figure 2d).

EDX was employed to analyze the composition of the AuNPs. The result clearly confirmed the presence of Au in both HB-AuNPs (Figure 2e) and MGF-AuNPs (Figure 2f). The peaks that correspond to Cu and C in the EDX spectra of the two AuNPs could be due to the carbon-coated copper grid, while the O in the HB-AuNPs could be from one of the phytochemicals present in the HBE, as previously argued by Wang et al. [37].

**Figure 3:**
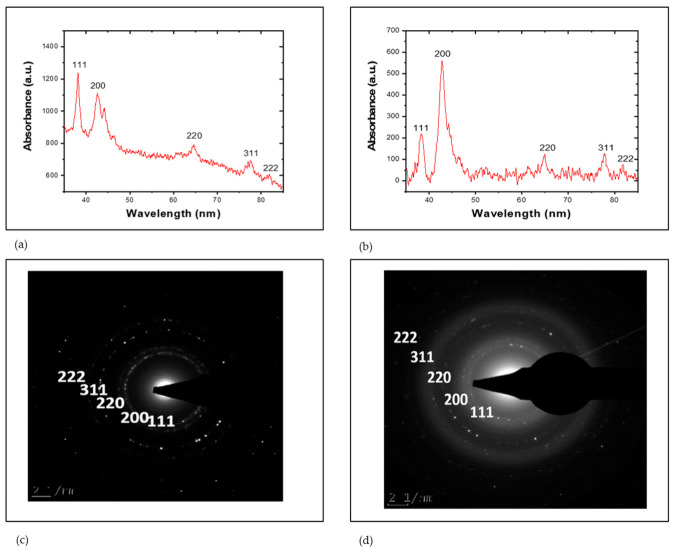
XRD and (SAED) patterns of the biogenic AuNPs as determined by XRD Spectroscopy and HR-TEM. XRD patterns of HB-AuNPs (**a**) and MGF-AuNPs (**b**); SAED patterns of HB-AuNPs (**c**) and MGF-AuNPs (**d**).

The XRD and Selective Area Electron Diffraction (SAED) patterns in Figure 3 demonstrated the crystalline nature of the HB-AuNPs and MGF-AuNPs. The XRD pattern for the two AuNPs revealed five peaks corresponding to standard Bragg’s reflection (111), (200), (220), (311) and (222) of face-centered cubic (fcc) lattice for AuNPs [38]. The more intense peaks correspond to (111) and (200) planes for HB-AuNPs (Figure 3a) and MGF-AuNPs (Figure 3b), which indicates the preferential growth of AuNPs at that orientation [32,39].

The SAED patterns of HB-AuNPs and MGF-AuNPs in Figure 3c,d respectively, also confirmed the appearance of ring-like peaks similar to the XRD fcc corresponding to (111), (200), (220), (311) and (222). The fcc nature of gold observed for HB-AuNPs and MGF-AuNPs gives a further clear indication that HB-AuNPs and MGF-AuNPs are composed of pure crystalline gold.

**Figure 4:**
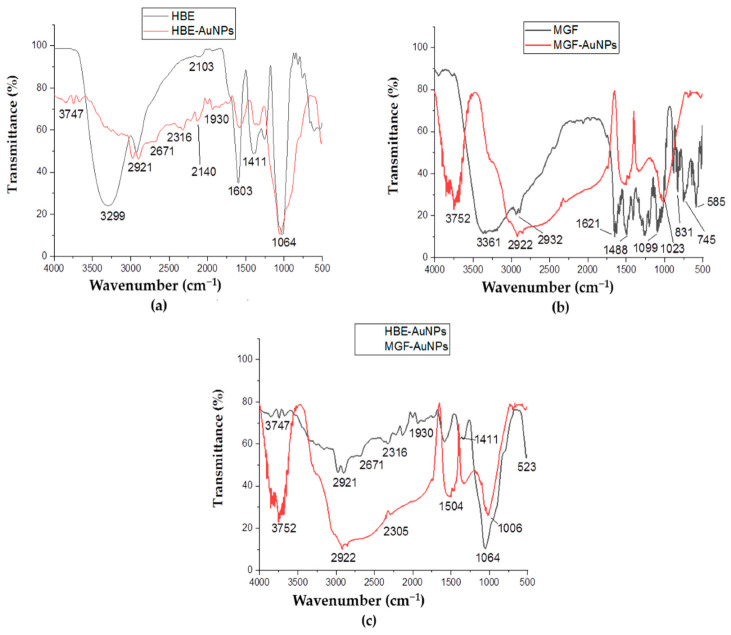
FTIR spectra of the HB and MGF extracts and their respective biogenic AuNPs. (**a**) HB-Extract and HB-AuNPs; (**b**) MGF-Extract and MGF-AuNPs; (**c**) HB-AuNPs and MGF-AuNPs.

The FTIR analysis was used to investigate the functional groups of the phytochemicals in HBE, MGF, HB-AuNPs and MGF-AuNPs. The FTIR spectra were compared to identify the functional groups of the phytochemicals present in HBE and MGF that were likely to be responsible for the reduction of gold ions to their zero-valence form. Figure 4a shows peaks at 3747 and 3299 cm^−1^ that are indicative of the presence of hydroxyl and amine groups, respectively. The O-H stretching is for alcohols that could be polyphenols and polysaccharides. The reduction in the intensity of peak at 3299 cm^−1^ in AuNPs is evidence of the participation of the hydroxyl functional groups. This takes place when alkoxide ions are generated that will then serve as a reducing agent [40]. According to Ovais et al., polyphenols are the chief reducing and/or capping agents in the plant-mediated green synthesis of metallic nanoparticles [41]. The bands at 2921 cm^−1^ for both the HBE and AuNPs, and an additional 2671 cm^−1^ for the latter are due to the C-H stretching of aromatic compounds. This provided further support for the presence of polyhydroxy aromatic compounds [31]. In both the HBE and the HB-AuNPs, the peak at 1411 cm^−1^ was due to bending vibration as a result of SP^2^ hybridization, which is common to alkenes and aromatic carbons [42]. The bands at 1064 cm^−1^ were observed for both the extract and the AuNPs corresponding to the C-O stretching of alcohols and carboxylic acids. This band is also assigned to the C-N stretching of aliphatic amines [43] mentioned earlier at 3747 cm^−1^ peak, thereby confirming the involvement of aliphatic amines in the formation of the HB-AuNPs. The weak bands at 2103 and 2140 cm^−1^ corresponded to C=N stretching, an indication that amides may also be involved in the synthesis of the HB-AuNPs [44].

The FTIR spectra of MGF and MGF-AuNPs are shown in Figure 4b. The 3362 cm^−1^ peak in the MGF is indicative of OH-alcohol stretching, its absence in the MGF-AuNPs suggests the involvement of the hydroxyl functional groups in the synthesis of MGF-AuNPs. In addition, the sharp peak at 2069 cm^−1^ corresponding to C-H stretching vibration in the FTIR spectra of MGF was significantly reduced in FTIR spectra of MGF-AuNPs. This further shows that C-H functional groups could also be involved in the reduction of gold ions [45]. Furthermore, the disappearance of peaks at 1488 and 1099 cm^−1^ corresponding to C-O-C and RCH_2_OH in MGF-AuNPs indicates the removal of the glucose unit from MGF [10]. Interestingly, these peaks are retained in HB-AuNPs indicating that the glucose units found in MGF might not be involved in the reduction and stabilization of HB-AuNPs. Overall, polyphenols, amines, polysaccharide, and amides were responsible for the formation of HB-AuNPs; while the hydroxyl and glucose units were involved in the formation of MGF-AuNPs. The FTIR spectra of HB-AuNPs and MGF-AuNPs showed some features that are quite similar as shown in Figure 4c. The FTIR suggests that the hydroxyl moieties that are also present in MGF were actively involved in the biosynthesis of HB-AuNPs.

### 2.2. In Vitro Stability of HB-AuNPs and MGF-AuNPs

**Figure 5:**
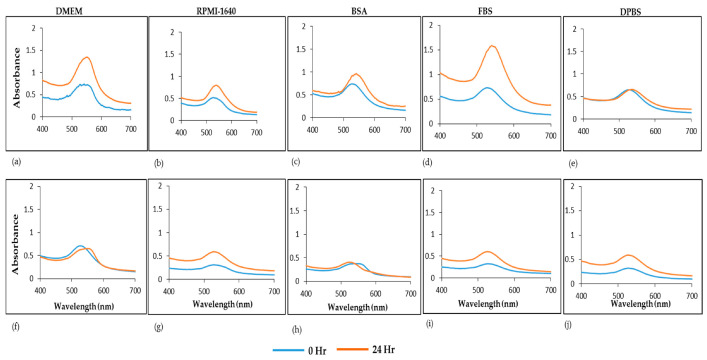
Stability of AuNPs in biological media at 0 and 24 h incubation at 37 °C. UV-Vis spectra of HB-AuNPs (**a**–**e**) and MGF-AuNPs (**f**–**j**) in DMEM (**a**,**f**), RPMI (**b**,**g**), BSA (**c**,**h**), FBS (**d**,**i**) and DPBS (**e**,**j**).

The stability of HB-AuNPs and MGF-AuNPs was evaluated at 0 and 24 h incubation in various biological media and buffers; these include Dulbecco’s Modified Eagle Medium (DMEM), Roswell Park Memorial Institute (RPMI 1640), Bovine serum albumin (BSA), fetal bovine serum (FBS) and Dulbecco’s Phosphate Buffered Saline (DPBS). The spectra indicating the presence of the AuNPs still remained following a 24 h incubation in various solutions, as depicted in Figure 5. However, a change in the SPR and their corresponding absorbances was observed especially for AuNPs in the two media (Figure 5a,f,b,g) and FBS (Figure 5d,i). HB-AuNPs showed an increase in absorbance (0.716 to 1.316) after 24 h incubation in DMEM (Figure 5a) while the MGF-AuNPs showed a moderate and negligible change in absorbance (0.642 to 0.63) and a redshift in the SPR (Figure 5f). These changes suggest that the AuNPs (especially HB-AuNPs) might be reacting with the components within the media and buffers, resulting in increased dispersion quality of the AuNPs leading to the observed changes [46]. In addition, the two AuNPs were found to be highly stable in water at room temperature.

### 2.3. Cytotoxicity of HB-AuNPs and MGF-AuNPs

HB-AuNPs and MGF-AuNPs were tested on cancer (Caco-2, U87 and PC-3) and non-cancer (MCF-12A) cell lines. The cells were treated with the nanoparticles (doses from 15.62 to 1000 µg/mL) and their viability was assessed by the colorimetric MTT assay after 24 h. The extracts did not show cytotoxicity on the four cell lines after 24 h treatment at the same concentrations (data not shown).

Of note, both HB and MGF have been reported to have antioxidant, antimutagenic and anti-cancer activities [47,48]. HB is known to improve the immune system, protect against inflammatory diseases, offer menopausal relief with phytoestrogenic effects, and have antimicrobial effects [48,49]. HB primarily exerts its anticancer effects through modulation of oxidative stress, inhibition of cell proliferation and inhibition of adenosine triphosphate production, all of which are closely associated with its high concentrations of monomeric polyphenols and flavonol compounds [49]. MGF on the other hand has been demonstrated using both in vitro and in vivo tests to have a broad spectrum effect on several types of cancers. Evidence suggests that MGF exerts its anticancer potential by downregulating inflammation, inhibiting cell cycle, offering protection against oxidative stress and DNA damage, enhancing apoptosis and inhibiting cell growth/invasion in malignant cells [13]. Studies have credited the pharmacological benefits of MGF-containing plants to MGF [12]. Intensive research has been done on *M. indica* L. as the main source of MGF to substantiate these claims. Interestingly, AuNPs synthesized from whole extracts and MGF from *M. indica* L. showed enhanced bio-activity when compared to the extracts alone.

**Figure 6:**
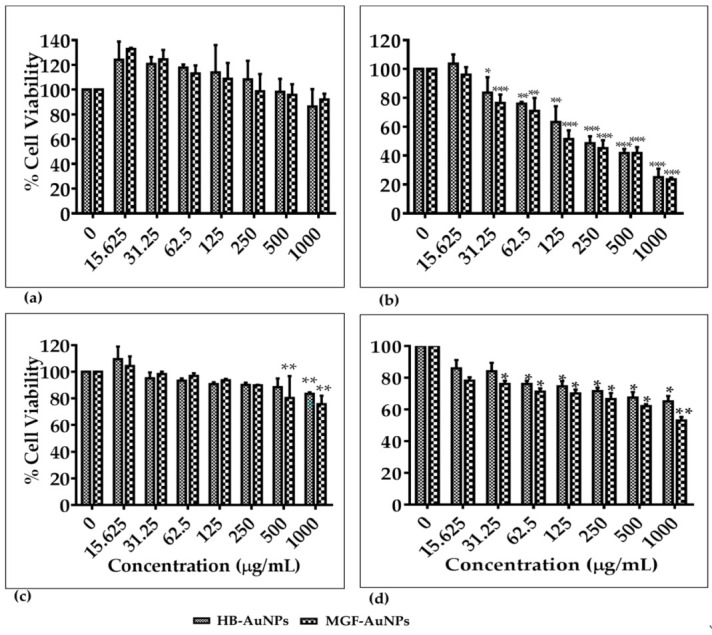
Investigation of the cytotoxic effects of HB-AuNPs and MGF-AuNPs in human cell lines. The in vitro effects of HB-AuNPs and MGF-AuNPs were tested in MCF-12A (**a**) U87 (**b**), Caco-2 (**c**) and PC-3 (**d**) cells. Data represented as the mean from three independent experiments. Error bars represent the standard error mean (± SEM). Data were considered to be statistically significant if *p* < 0.05, *** *p* < 0.001, ** *p* < 0.01, * *p* < 0.05.

The present study reports on the effect of AuNPs synthesized from HBE and MGF in cancer (Caco-2, U87 and PC-3) and non-cancer (MCF-12A) cells. The HB-AuNPs and the MGF-AuNPs showed similar toxicity toward the four cell lines as shown in Figure 6. The viability of MCF-12A cells was 100% or higher at all concentrations tested for both HB-AuNPs (15.62–250 µg/mL) and MGF-AuNPs (15.62–125 µg/mL) (Figure 6a). This suggests that the biogenic AuNPs at these concentrations seem to protect the cells and thus lead to an increment in their cell proliferation. The viability of MCF-12A cells was not significantly affected by either HB-AuNPs or MGF-AuNPs, even at high concentrations. However, both HB-AuNPs and MGF-AuNPs behaved differently on the cancer cells. Significant reduction in cell viability was observed in U87 (Figure 6b) and PC-3 (Figure 6d) cells at concentrations ranging from 31.25 to 1000 µg/mL, while the viability of Caco-2 was only affected at concentrations ranging from 500 to 1000 µg/mL (Figure 6c). These results suggest that the toxicity of both HB-AuNPs and MGF-AuNPs are fairly low in the Caco-2 cell lines compared to the other cell lines. The U87 cells were the most susceptible to the effects of MGF-AuNPs and HB-AuNPs. As summarized in Table 3, MGF-AuNPs and HB-AuNPs inhibited 50% (IC_50_ values) of U87 cell growth at 85.9 and 121.4 µg/mL, respectively, while a concentration higher than 1000 µg/mL was required to get IC_50_ values in the other three cell lines. Based on their cytotoxic profiles, HB-AuNPs and MGF-AuNPs have very similar biological activity. Both HB-AuNPs and MGF-AuNPs exhibited negligible cytotoxic effects on the MCF-12A cells even at the highest (1000 µg/mL) concentration. These results indicate that the two AuNPs might be biocompatible when used in vivo. Independent studies have reported the biocompatibility and low cytotoxic effects of biogenic AuNPs; MGF-AuNPs (up to 100 µM) were shown to be non-toxic on non-cancerous breast MCF-10A cells following a 24 h treatment [10]. Another study also reported that AuNPs synthesized from *Hibiscus sabdariffa* extracts had very little toxic effect in non-cancerous 293 cells [50]. The negligible toxicity of AuNPs in non-cancerous cells reported in the present study, in addition to earlier reports, further supports the preference of biogenic AuNPs for various biological applications. However, it should be noted that the four cell lines used in this study were derived from four different tissue origins. One of the limitations of this study was that the cancerous cell lines were not matched with a non-cancerous control cell lines of the same tissue type. While the non-cancerous MCF-12A cell line is derived from the breast tissue; the Caco-2, U87 and PC-3 cell lines were derived from the colon, brain, and prostate, respectively. As such, it is recommended that the cancer cell lines be matched with an appropriate non-cancerous cell line from the same tissue.

AuNPs have attracted significant interest over the last decades owing to their ease of synthesis, surface functionalization, chemical stability, biocompatibility, low toxicity and enhanced permeability and retention [51,52]. As a result, AuNPs are now widely researched for their potential in drug carrier/delivery, bioimaging, diagnosis and therapeutics. An earlier report demonstrated the significant anticancer effect of MGF-AuNPs against human PC-3 prostate cancer cells [6]. Khoobchandani et al. reported a 100% clinical benefit in breast cancer patients treated with biogenic AuNPs compared to standard anticancer drugs [9]. Resveratrol-AuNPs reduced cell viability of MDAMB-231, PANC-1 (pancreatic cancer cells) and PC-3 cancer cells in a dose-dependent (20–220 µg/mL) manner after treating for 24 and 48 h [53]. Consequently, the work presented here and previous studies describe promising results toward the integration of AuNPs, particularly of biological origin, in a cancer therapy regimen.

#### Co-Treatment of Caco-2 Cells with DOX and the Biogenic AuNPs

**Figure 7:**
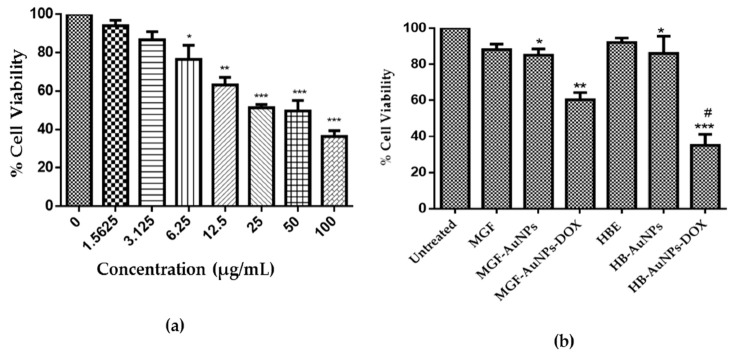
Co-treatment of Caco-2 cells with AuNPs and DOX enhanced cell death, compared to individual treatments. (**a**) shows the viability of Caco-2 cells following treatment with increasing concentrations (1.56–100 µg/mL) of DOX for 24 hrs. (**b**) shows the viability of Caco-2 cells following treatment with MGF (1000 µg/mL), HBE (1000 µg/mL), MGF-AuNPs (1000 µg/mL), HB-AuNPs (1000 µg/mL) and DOX (1.56 µg/mL) in combination with either MGF-AuNPs (MGF-AuNPs-DOX) or HB-AuNPs (HB-AuNPs-DOX). Data represented as the mean from three independent experiments. Error bars represent the standard error mean (± SEM). Data were considered to be statistically significant if *p* <0.05. *** *p* < 0.001, ** *p* < 0.01, * *p* < 0.05, # < 0.05 HB-AuNPs vs. MGF-AuNPs.

Due to their unique optical properties, AuNPs have many applications in medicine and can be used as drug delivery, photothermal, drug-sensitizing agents, among others. The drug-sensitizing effect of both HB-AuNPs and MGF-AuNPs was tested on the Caco-2 cells which showed resistance towards treatments with these AuNPs. The Caco-2 cells were treated with a combination of DOX and the AuNPs at least-toxic concentrations. As shown in Figure 7a, DOX showed a dose-response-induced cell death after 24 h treatment with 1.56–1000 µg/mL of the drug. DOX showed a significant cytotoxic effect on Caco-2 cells at higher concentrations (6.25–100 µg/mL), and negligible effects were observed at 1.56 µg/mL, which was selected for the co-treatment with the AuNPs.

After 24 h, the viability of cells treated with free DOX (1.56 µg/mL), HBE (1000 µg/mL), MGF (1000 µg/mL), MGF-AuNPs (1000 µg/mL) and HB-AuNPs (1000 µg/mL) reduced by ≤15% (Figure 7b). However, co-treatments with either DOX (1.56 µg/mL) and MGF-AuNPs (1000 µg/mL) or DOX (1.56 µg/mL) and HB-AuNPs (1000 µg/mL) further augmented the cytotoxic effect of DOX. While the viability of cells treated with DOX only or the AuNPs only reduced by about 10%, the viability of cells treated with DOX and MGF-AuNPs, or DOX and HB-AuNPs reduced by 40% and 70%, respectively. This data suggests that both MGF-AuNPs and HB-AuNPs appear to work in synergy with DOX to enhance the cytotoxic effect of DOX, and this effect was more pronounced on co-treatment with the HB-AuNPs.

In fact, DOX is a potent and valuable clinical anticancer agent; associated side effects and the development of resistance to DOX are the major problems limiting its use [54]. Cancers, especially colorectal cancers, are inherently resistant to DOX and require higher doses (as shown in this study) to effectively inhibit their growth. This consequently may result in adverse side effects including cardiotoxicity and nephrotoxicity [55]. Strategies that can sustain its efficacy while minimizing its off-target toxicity have been one of the major areas of research focus, and through co-treatment with metallic nanoparticles, this may be possible. An independent study reported the enhanced effect of DOX on human breast cancer (MCF-7) cells when used in combination with iron oxide nanoparticles [56]. In another study, the sensitivity of ovarian A2780 cancer cells to cisplatin was enhanced when used in combination with silver nanoparticles synthesized from curcumin [57]. The present study proposed that biogenic MGF-AuNPs and HB-AuNPs increased the sensitivity of Caco-2 colon cancer cells to a low concentration of DOX by offering a synergistic effect in inhibiting their growth. The findings from this study indicated that AuNPs synthesized from pure MGF and HBE that contains a substantial amount of MGF enhanced the efficacy of DOX.

## 3. Materials and Methods

### 3.1. Sample Preparation

MGF and GA powder were purchased from Sigma (St Louis, MO, USA). DOX was bought from Wuhan Sunrise Technology Development Company Limited (Wuhan, China). All these products were used as they were without any modification. Dried HB plant materials were donated to the Oxidative Stress Research Centre (Cape Peninsula University of Technology, South Africa) by Rooibos Ltd. (Clanwilliam, Western Cape, South Africa). The plant materials were stored in sealed plastic containers and kept at 25 °C in a dark room.

MGF, GA and DOX stock solutions were prepared in double distilled water. MGF and GA were prepared fresh. The HBE were prepared by boiling 100 g of the HB leaves in 1000 mL of double distilled water and kept on a magnetic stirrer without heat for 24 h. Afterwards, the infusion was filtered through Whatman No 1 followed by 0.45 µm filters. The resulting supernatant was freeze dried using FreeZone 25 L Freeze Dry (Labconco, Kansas City, MO, USA). The dried extracts were stored at 4 °C in sterile sealed containers for further use.

#### Phytochemical Analysis and Antioxidant Capacity

The aqueous HBE, MGF and GA (1 mg/mL) were investigated for the presence of flavanols, flavonols, total polyphenolic contents (TPCs) and antioxidant capacity, viz., ferric reducing antioxidant power (FRAP), oxygen radical absorbance capacity (ORAC) and 2,2-diphenyl-1-picrylhydrazyl (DPPH) using standard biochemical methods [58].

### 3.2. Synthesis of HB and MGF-AuNPs

HB-AuNPs were synthesized according to the previously described method [33] with few modifications. Briefly, 1 mL of the 2 mg/mL HBE dissolved in distilled water was mixed with 5 mL of 1 mM sodium tetrachloroaurate (III) dehydrate (Sigma Aldrich, St. Louis, MO, USA). The mixture was kept in a shaking incubator (40 rpm) for 1 h at 70 °C.

MGF-AuNPs were synthesized by a similar method and only differed in sample preparation and temperature used. Briefly, 4.2 mg of MGF (Sigma Aldrich) was dissolved in boiled 6 mL of double distilled water. To this mixture, 12 mg of GA powder was added as a stabilizer and the mixture was kept on a magnetic stirrer with constant stirring at 80 °C until MGF was completely dissolved. Then 0.2 mL of the MGF-GA mixture was added to 1 mL of warm 1 mM sodium tetrachloroaurate (III) dehydrate. Heat was switched off after the color changed to ruby red, and the mixture was continuously stirred on a heating block (200 rpm) for a further 60 min at room temperature.

#### 3.2.1. Characterization of the Biogenic AuNPs

The biogenic AuNPs were washed thrice in double distilled water and centrifuged at 14,000 rpm for 15 min, the AuNPs were resuspended in equal volume of double distilled water. The SPR of the AuNPs was determined using a POLARstar Omega microtitre plate reader (BMG Labtech, Offenburg, Germany) at a wavelength range of 400–800 nm. The hydrodynamic diameter, PDI and zeta potential of the AuNPs were determined using a Nano-ZS90 Zetasizer instrument (Malvern Panalytical Ltd, Enigma Business Park,, UK). A Perkin Elmer Spectrum Two Fourier transform infrared (FTIR) spectrophotometer (Waltham, MA, USA) was used to determine the functional groups in HBE, MGF, and their respective AuNPs. A high-resolution transmission electron microscope (TecnaiF20 HR-TEM, FEI Company, Hillsboro, OR, USA) linked with energy dispersive X-ray spectroscopy (EDX) was used to determine the morphology and crystalline nature of the AuNPs.

#### 3.2.2. Stability of the AuNPs in Biological Buffers

The in vitro stability of HB-AuNPs and MGF-AuNPs was determined by observing changes in the SPR band (UV-Vis spectra) following incubation in various biological media. A stability study was done by adding equal volumes of AuNPs with BSA, FBS, DPBS, DMEM supplemented with 10% FBS and 1% penicillin–streptomycin (pen-strep; Sigma) and RPMI 1640 supplemented with 10% FBS and 1% pen-strep following a previous method [33]. The absorbance of the mixture was read at 0 and 24 h using a POLARstar Omega microtitre plate reader.

### 3.3. Effects of Biogenic AuNPs on Non-Cancerous and Cancerous Cells

#### 3.3.1. Cell Culture

The human colon (Caco-2), prostate (PC-3), glioblastoma (U87) cancer and non-cancer breast (MCF-12A) cell lines were all purchased from the American Type Culture Collection (ATCC, Manassas, VA, USA). Caco-2 and U87 cells were maintained in DMEM (Gibco, Roche, Germany) supplemented with 10% FBS (Gibco) and 1% pen-strep (Gibco). PC-3 cells were maintained in RPMI 1640 (Gibco) supplemented with 10% FBS and 1% pen-strep. MCF-12A cells were maintained in DMEM/F12 supplemented with 10% FBS, 1% pen-strep, 10 µg/mL insulin (Gibco, Roche, Germany), 10 ng/mL epidermal growth factor (Sigma, St Louis, MO, USA) and 0.5 µg/mL hydrocortisone (Sigma). The cells were cultured in a humidified 5% CO_2_ incubator at 37 °C.

#### 3.3.2. Cell Viability Assay Using MTT Assay

The effect of the biogenic AuNPs and DOX on Caco-2, PC-3, U87, and MCF-12A cells was determined using MTT reagent following a previous protocol with slight modifications. The cells were individually seeded into sterile 96-well microtiter plates at a density of 1 × 10^5^ cells/mL and incubated at 37 °C for 24 h. Afterwards, the growth medium was replaced with 100 µL of either the AuNPs or DOX prepared in growth medium and further incubated for 24 h. Then, 10 µL of MTT reagent (5 mg/mL MTT) was added in each well and further incubated for 3 h at 37 °C. The MTT–medium mixture was replaced with 100 µL of dimethylsulfoxide (Kimix, Cape Town, South Africa) and the absorbance was measured on a POLARstar Omega microtitre plate reader at 570 nm and a reference wavelength of 700 nm. Cell viability was calculated in reference to the untreated (negative) control. The concentration of the AuNPs that inhibited 50% cell viability (IC_50_) in cancer cells was calculated using GraphPad Prism 6 software, access date 17/04/2020 [59,60].

The cell viability was also assessed for the Caco-2 cells treated with DOX and those co-treated with DOX and AuNPs. Here, the least toxic concentration of DOX on Caco-2 cells was evaluated by exposing the cells to increasing concentrations of DOX (1.56–100 µg/mL). This concentration was used for the co-treatment with AuNPs. Caco-2 cells were co-treated for 24 h with either DOX (1.56 µg/mL) and HB-AuNPs or DOX (1.56 µg/mL) and MGF-AuNPs (15.62 µg/mL) and the viability of the cells were assessed using the MTT assay as previously described.

## 4. Conclusions

This study shows for the first time the synthesis of AuNPs from an extract of *C. intermedia*. The study also shows a possible synergistic effect between DOX and AuNPs (HB-AuNP) produced from *C. intermedia*, as well as between DOX and AuNPs (MGF-AuNP) produced from MGF. The physicochemical characteristics and bioactivities of MGF-AuNPs and HB-AuNPs appear to be similar. Considering that HB-AuNPs were produced using an extract of *C. intermedia*, which contains a high concentration of MGF, it is possible that MGF is involved in the synthesis of HB-AuNPs. It is also likely that the synergistic effect between DOX and MGF-AuNPs, as well as DOX and HB-AuNPs can be ascribed to MGF, since several studies have suggested that MGF can enhance the anti-tumor effects of DOX. However, the mechanism of this synergistic effect needs to be investigated further and the involvement of MGF in the synthesis of HB-AuNPs from *C. intermedia* extract must be elucidated. The cytotoxic effects of this co-treatment must be investigated on a larger panel of cancer and normal cell lines to establish the selectivity of the treatment to specific cancers.

## Figures and Tables

**Table 1:** Phytochemical analysis and antioxidant capacity of HB extracts (HBE) and mangiferin (MGF).

Phytochemical Constituents	HBE	MGF
Flavanols (mg/g)	8.3753	−
Flavonols (mg/g)	0.3000	8.6742
Total phenolic content (TPC) (mgGAE/g)	0.1827	1.2992
2,2-diphenyl-1-picrylhydrazyl (DPPH) (µmolTE/g)	10.2601	75.3811
Oxygen radical absorbance capacity (ORAC) (µmolTE/g)	50.8520	376.2916
Ferric reducing antioxidant power (FRAP) (µmolAAE/g)	11.5828	94.8750

(−) = Not detected.

**Table 2:** Dynamic light scattering (DLS) analysis of HB-AuNPs and MGF-AuNPs.

AuNPs	Z-Average Size (nm)	PDI	Zeta Potential (mV)
HB-AuNPs	66.74 ± 9.7 nm	0.571 ± 0.01	−23.45 ± 1.4
MGF-AuNPs	65.50 ± 15.15 nm	0.432 ± 0.07	−27.87 ± 2.54

Results are expressed as mean ± SEM. PDI: polydispersity index.

**Table 3:** IC_50_ values of the AuNPs.

AuNPs	IC_50_ (µg/mL)			
	MCF-12A	U87	Caco-2	PC-3
HB-AuNPs	>1000	121.4	>1000	>1000
MGF-AuNPs	>1000	85.9	>1000	>1000

## Data Availability

The data presented in the study can be requested from the authors.

## Sample Availability

Samples of the AuNPs are available from the authors.
